# The ongoing challenge of large anal cancers: prospective long term outcomes of intensity-modulated radiation therapy with concurrent chemotherapy

**DOI:** 10.18632/oncotarget.24926

**Published:** 2018-04-17

**Authors:** Ali Hosni, Kathy Han, Lisa W. Le, Jolie Ringash, James Brierley, Rebecca Wong, Robert Dinniwell, Anthony Brade, Laura A. Dawson, Bernard J. Cummings, Monika K. Krzyzanowska, Eric X. Chen, David Hedley, Jennifer Knox, Alexandra M. Easson, Patricia Lindsay, Tim Craig, John Kim

**Affiliations:** ^1^ Radiation Medicine Program, Princess Margaret Cancer Centre, University Health Network, University of Toronto, Toronto, ON, Canada; ^2^ Department of Biostatistics, Princess Margaret Cancer Centre, University Health Network, Toronto, ON, Canada; ^3^ Department of Medical Oncology, Princess Margaret Cancer Centre, University Health Network, University of Toronto, Toronto, ON, Canada; ^4^ Department of Surgical Oncology, Princess Margaret Cancer Centre, University Health Network, University of Toronto, Toronto, ON, Canada

**Keywords:** anal cancer, chemoradiotherapy, IMRT, pattern of failure, outcomes

## Abstract

**Purpose:**

Patterns of failure and long term outcomes were prospectively evaluated following tumor factors-stratified radiation dose for anal/perianal cancer.

**Methods:**

Between 2008–2013, patients with anal/perianal squamous cell carcinoma were accrued to an institutional REB-approved prospective study. All patients were treated with image-guided intensity-modulated radiation therapy (IG-IMRT). Radiation dose selection (27–36 Gy for elective target, and 45–63 Gy for gross target) was based on tumor clinico-pathologic features. Chemotherapy regimen was 5-fluorouracil/mitomycin-C (weeks 1&5). Local [LF], regional failure [RF], distant metastasis [DM], overall- [OS], disease-free [DFS], colostomy-free survival [CFS] and late toxicity were analyzed.

**Results:**

Overall, 101 patients were evaluated; median follow-up: 56.5 months; 49.5% male; 34.7% T3/4-category, and 35.6% N+. Median radiation dose was 63 Gy. The most common acute grade ≥3 toxicities were skin (41.6%) and hematological (30.7%). Five-year OS, DFS, CFS, LF, RF, DM rates were 83.4%, 75.7%, 74.7, 13.9%, 4.6% and 5% respectively. Five-year LF for patients with T1-2 and T3-4 disease were 0% and 39.2% respectively. All LF (*n =* 14, after 63 Gy, in tumors ≥5 cm) were in the high dose volume except one marginal to the high dose volume. All RF (*n =* 4) were within elective dose volume except one within the high dose volume. On multivariable analysis, T3/4-category predicted for poor DFS, CFS and OS. The overall late grade ≥3 toxicity was 36.2% (mainly anal [20%]).

**Conclusions:**

Individualized radiation dose selection using IG-IMRT resulted in good long term outcomes. However, central failures remain a problem for locally advanced tumors even with high dose radiation (63 Gy/7weeks).

## INTRODUCTION

Although anal cancer is a relatively rare disease, its incidence is increasing [1, 2]. An estimated 40000 new cases were diagnosed worldwide in 2012, of which 88% were human papilloma virus (HPV) related [3]. Given its anatomical location and morbidity associated with surgical resection, the standard of care is sphincter-sparing chemoradiation. Local failure (LF) is the predominant pattern of relapse [4–7], emphasizing the importance of local control as a priority aim of treatment.

Phase III randomized controlled trials (RCTs) evaluated treatment intensification to improve the outcomes of anal cancer using induction chemotherapy [8–10], maintenance chemotherapy [11] and split-course higher dose (>60 Gy) radiation [10]. However, none of these strategies improved the outcomes. Moreover, patients with larger tumors had worse outcomes [8, 9], suggesting that a “one-size-fits-all” approach may not be appropriate.

A radiation dose-volume response has not clearly been defined. Data from RCTs are confounded by differences in proportion of T-category within the trials, the use and duration of planned mandatory radiotherapy (RT) gap, different overall radiation dose, and RT technique (2-dimensional versus 3-dimensional). There is limited data on patterns of failure and late toxicity in anal cancer [4, 8–11]. We prospectively evaluated the long term outcomes, pattern of failure and late toxicities of tumor clinico-pathologic features-based dose selection in anal/perianal cancer patients treated with image-guided intensity-modulated radiation therapy (IG-IMRT) and concurrent chemotherapy without a planned treatment break.

## RESULTS

### Patient and treatment characteristics

Between 2008 and 2013, 108 patients were consented, of whom 101 patients were evaluable for this analysis (7 were ineligible: two withdrew consent, two had carcinoma in situ, two had pre-treatment non-regional lymphadenopathy, and one patient was planned for surgery following chemoradiation). The median follow-up, censoring death, was 56.5 months (range, 14–87). Patient and tumor characteristics are summarised in Table 1. Ninety-five patients (94.1%) completed the planned radiation. The details of treatment characteristics and compliance are listed in Supplementary Table 1. The most common grade 3 acute toxicities were dermatitis (*n =* 40) and hematological (*n =* 22). Grade 4 acute toxicities were observed in 11 patients (10.9%) and included: neutropenia and/or leukopenia (*n =* 6), thrombocytopenia (*n =* 2), thrombocytopenia and dermatitis “perianal, inguinal and genital” (*n =* 1), perianal dermatitis (*n =* 1) and mitomycin pulmonary toxicity (*n =* 1). Five out of the 11 patients with grade 4 acute toxicity had human immunodeficiency virus (HIV) testing and all were HIV positive. There was no significant difference in the proportion of acute toxicities between patients who received ≤54 Gy (group A) versus >54 Gy (group B) except for more frequent dermatitis in group B (see Table 2).

**Table 1:** Patient and tumor characteristics

Characteristics	Whole cohort (*n =* 101)	Group A patients received ≤54 Gy (*n =* 43)	Group B patients received >54 Gy (*n =* 58)	Group A vs. group B *p* value
Age at diagnosis (years)				
Median (range)	57 (39–88)	54 (39–80)	57 (41–88)	0.19
Gender				
Male	50 (49.5%)	24 (55.8%)	26 (44.8%)	0.27
Female	51 (50.5%)	19 (44.2%)	32 (55.2%)	
HIV status				
Positive	25 (64.1%)	15 (68.2%)	10 (58.8%)	0.55
Negative	14 (35.9%)	7 (31.8%)	7 (41.2%)	
Not tested	62	21	41	
Anatomic subsite				
Anal canal only	70 (69.3%)	28 (65.1%)	42 (72.4%)	0.64
Anal canal with perianal extension	24 (23.8%)	11 (25.6%)	13 (22.4%)	
Perianal	7 (6.9%)	4 (9.3%)	3 (5.2%)	
Histologic grade				
High grade (G3)	19 (28.8%)	2 (7.7%)	17 (42.5%)	**0.0024**
Intermediate grade (G2)	25 (37.9%)	10 (38.5%)	15 (37.5%)	
Low grade (G1)	22 (33.3%)	14 (53.8%)	8 (20.0%)	
Not reported	35	17	18	
Maximum primary tumor size (cm),				
Median (range)	4 (1–20)	3 (1–8.6)^a^	5.3 (2–20)	**<0.0001**
T-category				
T1	11 (10.9%)	11 (25.6%)	0	**<0.0001**
T2	55 (54.5%)	30 (69.8%)	25 (43.1%)	
T3	28 (27.7%)	2 (4.7%) ^a^	26 (44.8%)	
T4	7 (6.9%)	0	7 (12.1%)	
N-category				
N0	65 (64.4%)	38 (88.4%)	27 (46.6%)	**0.0003**
N1	13 (12.9%)	1 (2.3%)	12 (20.7%)	
N2	17 (16.8%)	3 (7.0%)	14 (24.1%)	
N3	6 (5.9%)	1 (2.3%)	5 (8.6%)	
UICC/AJCC 7th edition stage grouping				
I	11 (10.9%)	11 (25.6%)	0	**<0.0001**
II	51 (50.5%)	27 (62.8%)	24 (41.4%)	
IIIA	15 (14.9%)	1 (2.3%)	14 (24.1%)	
IIIB	24 (23.8%)	4 (9.3%)	20 (34.5%)	

^a^Two patients with T3 (>5 cm) tumors were planned for 63 Gy and died on treatment: a) ischemic bowel (*n =* 1), and b) cardiac event while on treatment break for dermatitis (*n =* 1)

**Table 2:** Acute toxicity for anal cancer patients following chemoradiation with individualized radiation dose selection

Toxicity and grade	Whole cohort (*N =* 101)	Group A patients received ≤54 Gy (*N =* 43)	Group B patients received >54 Gy (*N =* 58)	Group A vs. group B *P* value
Hematologic worst acute toxicity				
Anemia				
1	60 (59.4%)	27 (62.8%)	33 (56.9%)	0.08
2	20 (19.8%)	6 (14.0%)	14 (24.1%)	
3	2 (2.0%)	0	2 (3.4%)	
Neutropnia				
1	19 (18.8%)	8 (18.6%)	11 (19.0%)	0.63
2	19 (18.8%)	8 (18.6%)	11 (19.0%)	
3	13 (12.9%)	6 (14.0%)	7 (12.1%)	
4	5 (5.0%)	1 (2.3%)	4 (6.9%)	
Leukopenia				
1	24 (23.8%)	14 (32.6%)	10 (17.2%)	0.61
2	26 (25.7%)	10 (23.3%)	16 (27.6%)	
3	20 (19.8%)	6 (14.0%)	14 (24.1%)	
4	3 (3.0%)	2 (4.7%)	1 (1.7%)	
Thrombocytopenia				
1	39 (38.6%)	14 (32.6%)	25 (43.1%)	0.44
2	9 (8.9%)	4 (9.3%)	5 (8.6%)	
3	7 (6.9%)	4 (9.3%)	3 (5.2%)	
4	3 (3.0%)	0	3 (5.2%)	
Gastrointestinal worst acute toxicity				
Nausea				
1	41 (40.6%)	16 (37.2%)	25 (43.1%)	0.21
2	8 (7.9%)	2 (4.7%)	6 (10.3%)	
Vomiting				
1	9 (8.9%)	4 (9.3%)	5 (8.6%)	0.09
2	6 (5.9%)	0	6 (10.3%)	
Diarrhea				
1	62 (61.4%)	29 (67.4%)	33 (56.9%)	0.36
2	28 (27.7%)	10 (23.3%)	18 (31.0%)	
3	4 (4.0%)	1 (2.3%)	3 (5.2%)	
Proctitis				
1	33 (32.7%)	14 (32.6%)	19 (32.8%)	0.48
2	56 (55.4%)	23 (53.5%)	33 (56.9%)	
3	3 (3.0%)	1 (2.3%)	2 (3.4%)	
Anal incontinence				
1	26 (25.7%)	11 (25.6%)	15 (25.9%)	0.32
2	6 (5.9%)	1 (2.3%)	5 (8.6%)	
Genitourinary worst acute toxicity				
1	62 (61.4%)	30 (69.8%)	32 (55.2%)	0.52
2	16 (15.8%)	4 (9.3%)	12 (20.7%)	
Skin worst acute toxicity				
Perianal				
1	8 (7.9%)	7 (16.3%)	1 (1.7%)	**0.003**
2	60 (59.4%)	27 (62.8%)	33 (56.9%)	
3	31 (30.7%)	9 (20.9%)	22 (37.9%)	
4	2 (2.0%)	0	2 (3.4%)	
Inguinal				
1	21 (20.8%)	14 (32.6%)	7 (12.1%)	
2	59 (58.4%)	23 (53.5%)	36 (62.1%)	**0.02**
3	20 (19.8%)	6 (14.0%)	14 (24.1%)	
4	1 (1.0%)	0	1 (1.7%)	
Genital				
1	19 (18.8%)	12 (27.9%)	7 (12.1%)	**0.048**
2	56 (55.4%)	24 (55.8%)	32 (55.2%)	
3	24 (23.8%)	7 (16.3%)	17 (29.3%)	
4	1 (1.0%)	0	1 (1.7%)	
Wight loss				
1	48 (47.5%)	23 (53.5%)	25 (43.1%)	0.58
2	29 (28.7%)	10 (23.3%)	19 (32.8%)	
Worst cardiac acute toxicity				
3	1 (1.0%)	0	1 (1.7%)	N/A^a^
Worst pulmonary acute toxicity				
3	1 (1.0%)	0	1 (1.7%)	N/A^a^
4	1 (1.0%)	0	1 (1.7%)	
Worst overall acute toxicity				
1	1 (1.0%)	0	1 (1.7%)	0.052
2	40 (39.6%)	22 (51.2%)	18 (31.0%)	
3	49 (48.5%)	19 (44.2%)	30 (51.7%)	
4	11 (10.9%)	2 (4.6%)	9 (15.5%)	

^a^Numbers were too small to be compared.

### Patterns of failure

**Figure 1:**
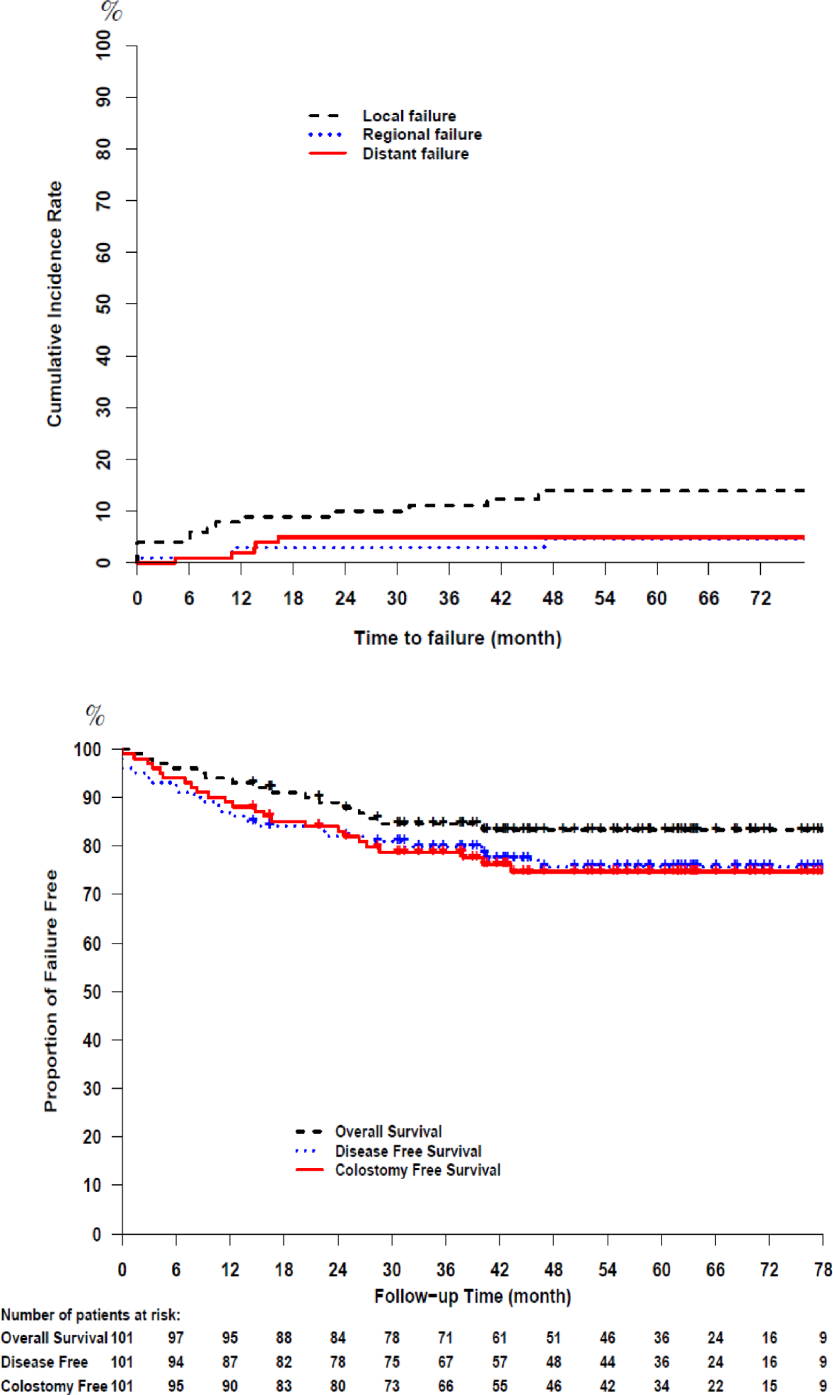
Long-term outcomes of chemoradiation with risk-stratified radiation dose selection

The estimated 5-year LF, regional failure (RF) and distant metastases (DM) rates were 13.9% (95% CI: 7.7%–22%), 4.6% (95% CI: 1.4%–10.7%), and 5% (95% CI: 1.8%–10.5%) respectively (Figure 1). Fourteen patients (7 anal canal, 1 perianal and 6 anal canal cancer with perianal extension) had LF at median (range) time of 9 (0–82) months. All LFs developed in patients with primary tumors ≥5 cm in maximum diameter who were treated with 63 Gy; in T2 (5 cm) disease (*n =* 1), T3 (*n =* 11) and T4 (*n =* 2), (see Supplementary Table 2). All LFs were high dose central failures except for one patient with progressive disease after IMRT who had a high dose marginal failure. Salvage abdominoperineal resection (APR) was performed in 50% (7 out of 14) of patients who had LF, but not undertaken in others due to: locally unresectable disease (*n =* 3), patient preference (*n =* 2), synchronous DM (*n =* 1) and concurrent non-small-cell lung cancer (*n =* 1). Among the patients who underwent salvage APR, five remained disease free until the last follow up, while two patients had DM after the surgery.

**Figure 2:**
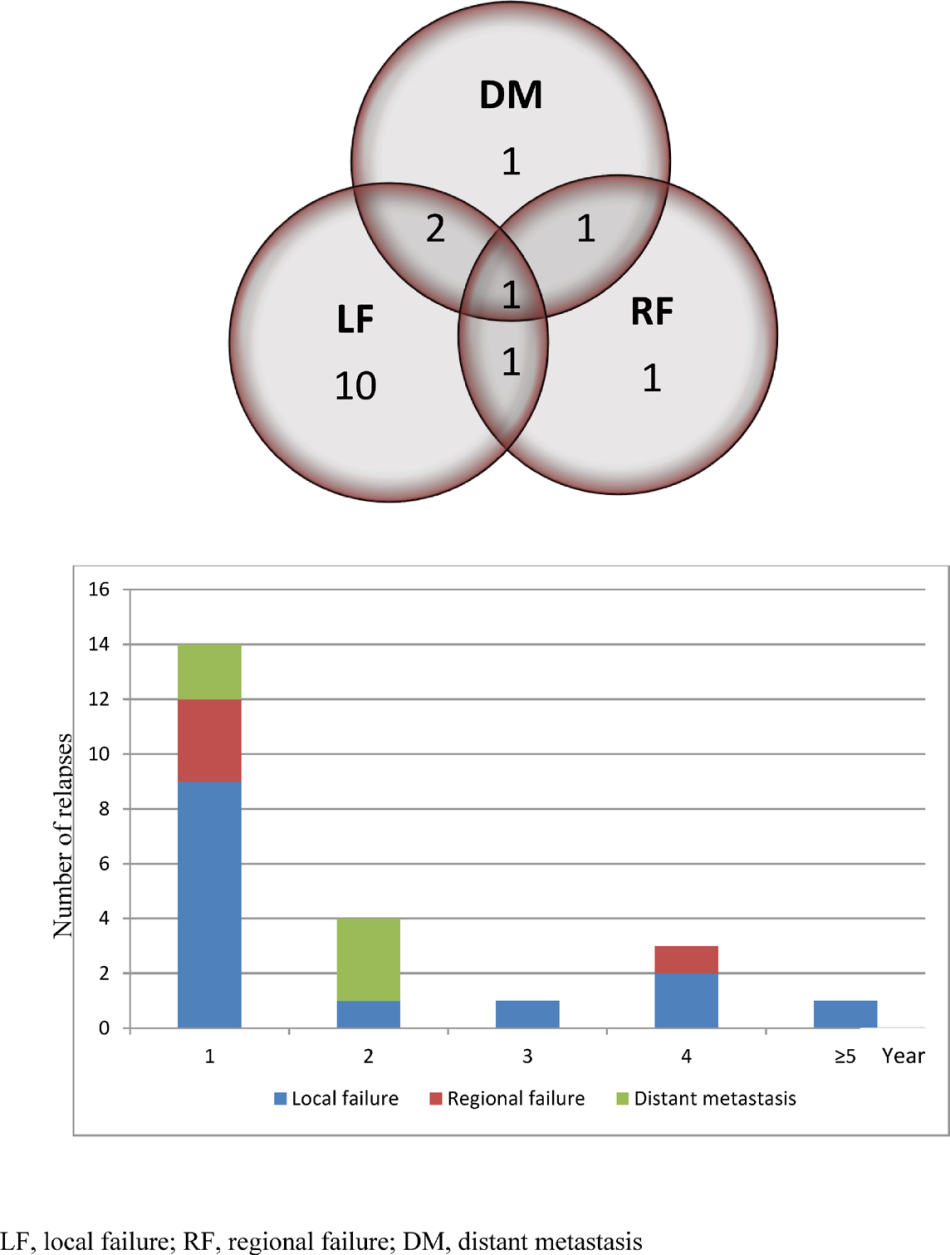
Distribution and pattern of failure in anal/perianal cancer

Four patients developed RF in N0 (*n =* 2), N1 (*n =* 1) and N2 disease (*n =* 1), at median 11 months (range, 0–48). One patient who had perirectal RF with synchronous LF refused salvage APR and was treated with palliative RT. One patient with isolated inguinal RF underwent inguinal lymph node dissection. Two patients had inguinal and external iliac RF plus non-regional nodal failure and were treated with palliative RT. The perirectal RF (*n =* 1) was classified as a high dose central failure, while all inguinal and external iliac RF (*n =* 3) were elective dose central failures. Eight (24.2%) out of 33 patients who had RT interruption due to acute toxicity developed locoregional failure (LRF). The distribution and patterns of failure are presented in Figure 2.

Five patients had DM at a median (range) of 13 (4–16) months, of whom 2 developed non-regional nodal metastasis (common iliac +/– paraortic) with synchronous inguinal and external iliac RF, while 2 patients had lung metastases 6 months after salvage APR for LF, and 1 patient was diagnosed with pathologically-proven kidney metastasis followed by extensive non-regional (para-aortic and mediastinal) lymphadenopathy, while his primary tumor and regional nodes were controlled. Non-regional nodal failures in the common iliac region (*n =* 2) were elective dose marginal (*n =* 1) and extraneous (*n =* 1) failures. Three patients with DM were treated with chemotherapy; however all of them died within 1 year from the diagnosis of DM.

### Survival

A total of 17 patients died. Two patients died on treatment from: a) ischemic bowel (*n =* 1), and b) cardiac event while on treatment break for dermatitis (*n =* 1). Other causes of death included anal/perianal cancer (*n =* 9), lung and recurrent perianal cancer (*n =* 1), and metastatic penile cancer (*n =* 1), while 3 patients died of unknown cause. The 5-year overall- (OS), disease-free (DFS), colostomy-free survival (CFS) were 83.4% (95% CI: 76.2–91.2), 75.7% (95% CI: 67.5–85%) and 74.7 (95% CI: 66.4%–84.1%) respectively (Figure 1). Colostomy was performed in 15 patients; 2 planned temporary colostomy for bowel obstruction prior to start of IMRT (of whom, one had a successful reversal), 9 after LF (7 as part of salvage APR and 2 palliative), 1 due to perianal sepsis secondary to LF (persistent anal carcinoma) and treatment complication (radiation-induced necrosis); and 3 due to treatment complications: incontinence (*n =* 2), and bowel perforation (*n =* 1).

### Predictors of outcome

Factors evaluated by univariable analysis are shown in Table 3. Multivariable analysis identified T3-4 category as predictor of poor DFS (HR = 7, 95% CI: 2.8–17.8, *p <* 0.001), CFS (HR = 3.7, 95% CI: 1.6–8.4, *p =* 0.002) and OS (HR = 5, 95% CI: 1.7–14.7, *p =* 0.004). Male gender was associated with poor DFS and OS, while tumor location (anal canal with perianal extension) was prognostic for lower CFS, and older age predicted for inferior OS (Table 3). Because 63 Gy was prescribed exclusively in advanced disease, a meaningful statistical analysis of the relationship between dose and outcome was not possible.

**Table 3:** Univariable and multivariable analyses of prognostic factors of overall, disease free- and colostomy free- survival

Variable	Overall survival				Disease free survival				Colostomy free survival			
	Univariable		Multivariable		Univariable		Multivariable		Univariable		Multivariable	
	**HR** **(95% CI)**	***P*** **value**	**HR** **(95% CI)**	***P*** **Value**	**HR** **(95% CI)**	***P*** **value**	**HR** **(95% CI)**	***P*** **value**	**HR** **(95% CI)**	***P*** **value**	**HR** **(95% CI)**	***P*** **value**
T3-4 category	3.59 (1.30–9.88)	0.01	4.98 (1.69–14.72)	**0.004**	6.85 (2.70–17.40)	0.001	7.02 (2.76–17.83)	**0.001**	3.83 (1.68–8.77)	0.002	3.65 (1.59–8.37)	**0.002**
Male gender	3.38 (1.09–10.50)	0.04	4.50 (1.42–14.27)	**0.01**	2.33 (1.001–5.46)	0.05	2.46 (1.04–5.73)	**0.04**	2.00 (0.88–4.54)	0.10	–	–
Age ^a^	1.05 (1.001–1.09)	0.045	1.05 (1.002–1.09)	**0.04**	1.03 (0.99–1.07)	0.12	–	–	1.02 (0.99–1.06)	0.23	–	–
Anal canal cancer with perianal extension	3.04 (1.10–8.38)	0.03	–	–	2.92 (1.26–6.75)	0.01	–	–	3.47 (1.56–7.74)	0.002	3.17 (1.42–7.09)	**0.005**
N+ category	1.77 (0.57–5.47)	0.33	3.28 (0.98–10.90)	0.054	1.09 (0.47–2.56)	0.84	–	–	0.93 (0.41–2.1)	0.85	–	–
High histological grade	1.68 (0.47–5.96)	0.42	–	–	2 (0.74–5.37)	0.17	–	–	1.77 (0.67–4.67)	0.25	–	–
Maximum primary tumor size^a^	1.16 (1.02–1.32)	0.02	–	–	1.23 (1.12–1.34)	0.001	–	–	1 .18 (1.08–1.29)	0.0003	–	–
RT interruption, days^a^	1 (0.92–1.09)	0.99	–	–	1.01 (0.95–1.08)	0.71	–	–	1.05 (1–1.11)	0.054		

^a^Continuous variable.

RT, radiation therapy.

### Late toxicity:

Late toxicities were mainly grade 1 or 2 (Table 4). The most common type of late grade 3 or 4 toxicity was anal (20%, 95% CI: 13%–30%). Twelve patients had grade 4 toxicities: rectovaginal fistula (*n =* 2), rectal perforation (*n =* 1), anal incontinence requiring colostomy (*n =* 2), perianal skin ulceration (*n =* 4), and fracture of the sacrum and/or symphysis pubis (*n =* 4). In comparison between late toxicity in group A (patients received ≤54 Gy) and group B (patients received >54 Gy), only late anal toxicity was more frequent in group B; this was mainly due to a higher proportion of grade 2 anal toxicity in group B (see Table 4), with no association between dose >54 Gy and grade ≥3 late anal toxicity (HR: 1.8, 95% CI: 0.62–5.23, *p =* 0.28).

**Table 4:** Late toxicity for anal cancer patients following chemoradiation with individualized radiation dose selection

Toxicity and grade	Whole cohort 94 out of 101^a^	Group A patients received ≤54 Gy 40 out of 43 ^a^	Group B patients received >54 Gy 54 out of 58 ^a^	Group A vs. group B *P* value
Intestinal worst late toxicity				
1	35 (37.2%)	16 (40%)	19 (35.2%)	0.19
2	37 (39.4%)	14 (35%)	23 (42.6%)	
3	0	0	0	
4	2 (2.1%)	0	2 (3.7%)	
Anal worst late toxicity				
1	22 (23.4%)	12 (30.0%)	10 (18.5%)	**0.04**
2	33 (35.1%)	11 (27.5%)	22 (40.7%)	
3	17 (18.1%)	6 (15.0%)	11 (20.4%)	
4	2 (2.1%)	0	2 (3.7%)	
Bladder worst late toxicity				
1	7 (7.4%)	5 (12.5%)	2 (3.7%)	1.00
2	13 (13.8%)	6 (15.0%)	7 (13.0%)	
3	1 (1.1%)	0	1 (1.9%)	
4	1 (1.1%)	0	1 (1.9%)	
Skin worst late toxicity				
1	37 (39.4%)	22 (55.0%)	15 (27.8%)	0.30
2	39 (41.5%)	11 (27.5%)	28 (51.9%)	
3	4 (4.3%)	0	4 (7.4%)	
4	4 (4.3%)	3 (7.5%)	1 (1.9%)	
Bone worst late toxicity				
1	6 (6.4%)	2 (5.0%)	4 (7.4%)	0.62
2	7 (7.4%)	3 (7.5%)	4 (7.4%)	
3	2 (2.1%)	1 (2.5%)	1 (1.9%)	
4	4 (4.3%)	1 (2.5%)	3 (5.6%)	
Erectile dysfunction worst late toxicity^b^				
1	11 (23.4%)	7 (30.4%)	4 (16.7%)	1.00
2	16 (34.0%)	9 (39.1%)	7 (29.2%)	
3	5 (10.6%)	1 (4.3%)	4 (16.7%)	
Dyspareunia worst late toxicity^b^				
1	9 (19.1%)	5 (29.4%)	4 (13.3%)	0.17
2	8 (17.0%)	5 (29.4%)	3 (10.0%)	
3	8 (17.0%)	3 (17.6%)	5 (16.6%)	
Overall worst late toxicity				
1	16 (17%)	10 (25%)	6 (11.1%)	0.13
2	42 (44.7%)	17 (42.5%)	25 (46.3%)	
3	22 (23.4%)	8 (20%)	14 (25.9%)	
4	12 (12.8)	4 (10%)	8 (14.8%)	

^a^Late toxicity was assessed in 94 out of 101 patients (93%); of whom 40 received ≤54 Gy and 54 received >54 Gy.

^b^Erectile dysfunction was assessed in 47 males; 23 in group A and 24 in group, while dyspareunia was assessed in 47 females: 17 in group A and 30 in group B.

## DISCUSSION

This prospective study evaluated the treatment of anal cancer patients with continuous course IG-IMRT, with radiation dose selection based on predefined risk stratification. To our knowledge, this is the largest reported prospective IG-IMRT cohort for anal cancer. The results are favorable compared to previous studies which used non-IMRT techniques [4, 8–11], and recent phase I-II studies which used lower radiation dose prescription [12, 13]. However, locally advanced anal tumors remain a challenge, with more than one third of them failing locally.

In this study, we reported an overall grade ≥3 acute toxicity (59.4%) somewhat lower than prior reports. The overall grade ≥3 acute toxicity in previous RCTs (using non-IMRT techniques) ranged from 71% to 87% [8, 11], and in the RTOG 0529 prospective phase II IMRT study, this rate was 83% [14]. In addition to IMRT technique [15], individualized radiation dose selection, standardized target volume delineation and planning, and daily image guidance may be contributing factors for improving the acute toxicity outcome. Moreover, patients in group B (who received >54 Gy) had more frequent acute skin toxicity given the higher radiation dose to the gross primary tumor (contributing to perianal and genital skin toxicity [especially with perianal tumor extension]), and the higher incidence of inguinal lymph node involvement in group B compared to group A (21% vs. 7%) with subsequent use of higher radiation dose to the gross nodal disease (contributing to both inguinal and genital skin toxicity), see Supplementary Table 2. Notably, the coverage for gross (primary and nodal) target volume has higher priority than genital sparing to avoid potential consequence of marginal miss.

Many uncertainties remain in the RT break during the management of anal cancer. Earlier RCTs advocated a mandated 4 to 6-week gap in RT after 45 Gy [4, 6, 7]. In the ACT II RCT, patients were treated with 50.4 Gy with no planned gap, however 13% of them had a treatment break in view of acute toxicity [11]. The RTOG 9811 RCT did not also incorporate a gap, with a higher total RT dose (55–59 Gy) was prescribed, and the RT breaks were reported in 62% of patients [8, 9]. Despite that the patients in the RTOG 0529 phase II study were treated with IMRT to a total RT dose of 50.4 to 54 Gy, the treatment breaks due to toxicity were needed in 49% [14]. In our study, treatment interruption due to acute toxicity was reported in 33% of the whole cohort, and more frequently among patients in group B (41%). Moreover, on univariable analysis, the duration of treatment break was not associated with poor outcomes in our cohort. Such observations require cautious interpretation particularly with the lack of randomized data and the contradicting results of retrospective analyses regarding the impact of treatment interruption on the outcomes for anal cancer [16–18]. In accordance with the radiobiological principles, the whole RT course should be completed with the avoidance of split course treatment and keeping the RT breaks as short as possible.

Late toxicity data following anal cancer treatment is sparse. While we used continuous course IMRT with a median total radiation dose of 63 Gy, our observed grade ≥3 late toxicities were comparable to those from RTOG 9811 RCT (which used continuous course RT with a total dose less than 60 Gy) [8, 9] and ACCORD 03 RCT (which used a total radiation dose above 60 Gy but with a planned RT gap of 3 weeks after 45 Gy) [10].

The majority of treatment failures (mainly LF) were observed in the first 2 years following IMRT, with 3- and 5-year cumulative incidence LF rates of 11.1% and 13.9% respectively. A dose range of 45 to 63 Gy was able to control the small primary tumor locally with a 5-year LF of 0% for patients with T1-2 disease, while high dose radiation (63 Gy) did not achieve the same successful results in large tumors with a 5-year LF of 39.2% in patients with T3-4 disease which represent approximately one third of our cohort (see Supplementary Table 3). Therefore, and in consistent with previous RCTs [9, 11], T3-4 category predicted for poor OS, DFS and CFS. Moreover, all but one LF were high dose central failure (one marginal failure with progressive disease), indicating the need for further treatment intensification of this subgroup.

Induction [8–10] and maintenance chemotherapy [11] have failed to improve the outcomes of anal cancer. The addition of cetuximab to chemoradiotherapy resulted in a 3 year LRF of approximately 20% in two prospective phase II studies: the E3205 study (54% of patients had T3-4 disease, and overall G3-4 acute toxicity was 87%)[12], and the AMC045 study (27% of patients had T3-4 disease, and overall G3-4 acute toxicity was 72%) [13]. The final results of the VITAL trial (NCT01285778: panitumumab combination with chemoradiotherapy) and Brown University study (NCT01671488: listeria monocytogens listerolysin-O immunotherapy with chemoradiotherapy) are eagerly awaited.

Our study has some limitations, including the non-randomized nature and the lack of data regarding HPV status including integration of HPV status in the risk stratification for individualized radiation dose selection and its correlation with the outcomes. Additionally, sample size was limited due to conduct of the study at a single large academic center; international collaboration should be considered for future studies. Nonetheless, this is the first prospective study describing the pattern of failure of anal cancer in the era of IMRT. The unique selection of radiation dose according to the predefined risk category has resulted in favourable long term outcomes in tumors <5 cm. However, the challenge remains to improve local control for T3-4 disease. Combination of targeted agents or immunotherapy with chemoradiation could be possible options. Advances in imaging and radiotherapy techniques present potential opportunities for exploration of further RT dose escalation to the gross target volume in high risk patients, and possibly enabling dose reduction in other patients.

In conclusion, the individualized radiation dose selection using IG-IMRT resulted in good long term outcomes with acceptable toxicity. However, central failures remain a problem for locally advanced tumors even with high dose radiation (63 Gy/7weeks).

## MATERIALS AND METHODS

### Study design and participants

This prospective study evaluated patients with primary non-metastatic (M0) anal canal and perianal histologically-confirmed invasive squamous cell carcinoma treated with curative IG-IMRT with or without concurrent chemotherapy. Patients with history of, or contraindication to, pelvic radiotherapy were excluded. The study protocol was approved by the local institutional research ethics board and all patients provided written consent and were prospectively treated following the study guidelines.

### Treatment

All patients underwent CT and MRI simulation in a prone position with a full bladder. The high dose clinical target volume was generated by expanding the gross tumor volume (primary or nodal) with a 5 mm margin in all directions, with exclusion of the air, uninvolved bone, muscle or any tissues not at risk for microscopic spread. The elective nodal clinical target volume included the external iliac, internal iliac, presacral, perirectal and inguinal nodal regions, with the most cranial aspect of the elective nodal volume corresponding to the bifurcation of the common iliac vessels into external/internal iliacs (approximate boney landmark: sacral promontory).

All RT plans were two-phase sequential boost IG-IMRT technique to maintain 1.8 Gy/fraction. Patients were allocated into three categories based on clinico-pathologic features: 1) T1N0 tumor (radiation dose prescription was 27 Gy/15 fractions to elective target and 45 Gy/25 fractions to gross target), 2) T1N+ or T2 with low/intermediate grade tumor less than 4 cm (36 Gy/20 fractions to elective target and 54 Gy/30 fractions to gross target), and 3) T2 (if high grade or ≥4 cm) and T3-4 tumors (36 Gy/20 fractions to elective target and 63 Gy/35 fractions to gross target). The chemotherapy regimen was 5-fluorouracil and mitomycin-C during weeks 1 and 5. Treatment was guided by institutional standards: treatment policies, contouring, planning and dose-volume criteria, with peer review quality assurance and daily bone match cone beam CT image guidance. The details of IMRT and chemotherapy components of treatment have been previously described [15].

### Evaluation

Staging was conducted in accordance with AJCC/UICC 7th edition following digital rectal examination, MRI of the pelvis and CT of the chest, abdomen and pelvis. HIV testing was limited to patients who deemed at risk, and all patients followed the same treatment protocol regardless the HIV status. Patients were assessed weekly during treatment. Initial post-treatment imaging evaluation was performed 10–12 weeks following IMRT. Patients were seen monthly during the first 3 months, every 3 months for 2–3 years, every 6 months until 5 years, and then annually. Follow up endoscopic evaluation was performed as clinically indicated.

### Data collection

Acute toxicity data were collected within 3 months from the start of IMRT, while late toxicity data were defined after 3 months following the start of IMRT. All acute toxicities were graded according to the National Cancer Institute Common Terminology Criteria for Adverse Events (NCI-CTCAE), version 3.0, except for acute skin and genitourinary toxicities which were graded according to the RTOG acute radiation morbidity score. Radiation was interrupted at the discretion of treating physician. Typically, the treatment was held for grade 4 or non-tolerable grade 3 acute toxicity, until the toxicity was reduced to grade ≤3 and the patient was able to tolerate the RT. Late toxicities were graded according to RTOG/EORTC late radiation morbidity score, except erectile dysfunction and dyspareunia (which were graded based on NCI-CTCAE, version 3.0), and late anal toxicity (which was graded with specific criteria described by John *et al.* [19]).

Tumor control and survival were determined at each follow-up visit. To analyze patterns of failure, the recurrent gross tumor volume (rGTV) was contoured on the planning CT after registration/fusion with the first diagnostic scan showing recurrence. The pattern of failure was defined according to the dose prescription into high- and elective- dose recurrence, and further sub-classified into: “central failure” if 95% of rGTV was within the 95% isodose of the intended treatment dose, ‘‘marginal failure’’ if 20% to <95% of rGTV occurred within the 95% isodose, and ‘‘extraneous failure’’ if <20% of rGTV occurred within the 95% isodose [20, 21]. While patients with pre-treatment external iliac lymphadenopathy were excluded from the study (as they were classified as metastatic disease at the time of study recruitment according to UICC/AJCC 7th edition), external iliac nodal involvement is now considered regional disease (N-category) based on UICC/AJCC 8th edition; thus we classified external iliac nodal failure as regional failure for the purpose of analysis.

### Statistical considerations

Acute and late toxicity rates were estimated using proportion and associated binomial 95% CI and were compared between patients who received ≤54 Gy (group A) and >54 Gy (group B) using the Cochran–Armitage trend test. Local failure (LF), regional failure (RF) and distant metastasis (DM) rates were estimated using the cumulative incidence method, with death as a competing risk. Colostomy-free (CFS), disease-free (DFS) and overall survival (OS) were analyzed using the Kaplan-Meier method. Outcomes were calculated from the first day of IMRT. Multivariable analysis (MVA) using Cox proportional hazards regression was used to identify predictors of DFS, CFS and OS. All reported *p* values were 2-sided, with a statistical significance level of *p* < 0.05.

## SUPPLEMENTARY MATERIALS TABLES


