# Supplementary material for: The ongoing challenge of large anal cancers: prospective long term outcomes of intensity-modulated radiation therapy with concurrent chemotherapy
Source: Oncotarget. 2018 Apr 17;9(29):20439–50. doi: 10.18632/oncotarget.24926 (PMC5945520; doi:10.18632/oncotarget.24926)
Supplement: Supplementary file 1 [file oncotarget-09-20439-s001.pdf]

The ongoing challenge of large anal cancers: prospective long term outcomes of intensity-modulated radiation therapy with concurrent chemotherapy

SUPPLEMENTARY MATERIALS

Supplementary Table 1: Treatment characteristics and compliance

	Whole cohort (*n* = 101)	Group A patients received ≤54 Gy (*n* = 43)	Group B patients received >54 Gy (*n* = 58)
Total radiotherapy prescription dose			
Planned 63 Gy	60 (92%)	2[a]	58[b]
Planned 54 Gy	35 (97%)	35[c]	–
Planned 45 Gy	6 (100%)	6	–
Concurrent chemotherapy regimen			
2 cycles of mitomycin-5Fu	85/101 (84.2%)	38/43 (88.4%)	47/58 (81.0%)
Dose reduction	19/85 (22.4%)	8/38 (21.1%)	11/47 (23.4%)
Single cycle of mitomycin-5Fu	8/101 (7.9%)	4/43 (9.3%)	4/58 (6.9%)
Dose reduction	3/8 (37.5%)	0	3/4 (75.0%)
2 cycles of cisplatin-5Fu[d]	1/101 (1.0%)	0	1/58 (1.7%)
2 cycles of 5Fu only[d]	4/101 (4.0%)	0	4/58 (6.9%)
No chemotherapy	3/101 (3.0%)	1/43 (2.3%)	2/58 (3.4%)
Radiotherapy interruption due to acute toxicity causes[e]:	33 (32.7%)	9 (21%)	24 (41%)
Dermatitis	28 (27.7%)	9 (20.9%)	19 (32.8%)
Proctitis	2 (2.0%)	1 (2.3%)	1 (1.7%)
Nausea/vomiting	2 (2.0%)	0	2 (3.4%)
Diarrhea	2 (2.0%)	0	2 (3.4%)
Neutropenia	3 (3.0%)	0	3 (5.4%)
Thrombocytopenia	1 (1.0%)	0	1 (1.7%)
Acute coronary syndrome	1 (1.0%)	0	1 (1.7%)
Pulmonary embolism	1 (1.0%)	0	1 (1.7%)
Mitomycin-C pulmonary toxicity	1 (1.0%)	0	1 (1.7%)
Timing to treatment break Median (range), radiotherapy fractions[f]	24 (9–30)	23 (16–26)	24 (9–30)
Duration of treatment break Median (range), days[f]	8 (1–25)	5 (1–10)	9 (1–25)
RT interruption < 8 days[f]	14/33 (42.4%)	7/9 (77.8%)	7/24 (29.2%)
RT interruption ≥ 8 days[f]	19/33 (57.6%)	2/9 (22.2%)	17/24 (70.1%)
Overall treatment time Median (range), days	50 (29–88)	43 (29–60)	52 (47–88)

[a]Two patients were planned for 63 Gy and died on treatment in view of: a) ischemic bowel (*n* = 1), and b) cardiac event while on treatment break for dermatitis (*n* = 1)
[b]All received the planned 63 Gy except 1 received 61.2 Gy and 2 received 59.4 Gy
[c]All received 54 Gy except 1 received 50.4 Gy
[d]No dose reduction of the used regimen
[e]Each patient may have >1 cause
[f]Only for patients who had treatment interruption due to acute toxicity

Supplementary Table 2: Frequency of sever inguinal and genital skin acute toxicity

	Group A patients received ≤54 Gy (*n* = 43)		Group B patients received >54 Gy (*n* = 58)	
	No inguinal LN involvement (*n* = 40)	Inguinal LN involvement (*n* = 3)	No inguinal LN Involvement (*n* = 46)	Inguinal LN involvement (*n* = 12)
Grade ≥3 inguinal skin toxicity	6 (15%)	0	10 (16%)	5 (42%)
Grade ≥3 genital skin toxicity	6 (15%)	1 (33%)	12 (26%)	4 (33%)

LN, lymph node.

Supplementary Table 3: Local failure according to the pre-defined clinic-pathologic features

	**Whole cohort** ***n*** **= 101**	**T1N0** ***n*** **= 11**	**T1N+ or T2 < 4 cm** ***n*** **= 27**	**T2 ≥ 4 cm** ***n*** **= 28**	**T3-4** ***n*** **= 35**
Prescribed dose to elective target (Gy)	27–36	27	36	36	36
Prescribed dose to gross target (Gy)	45–63	45	54	63	63
3-year local failure	11.1% (95% CI: 5.8–18.2%)	0%	0%	0%[a]	31.7% (95% CI: 17.0–47.5%)
5-year local failure	13.9% (95% CI: 7.7–22.0%)	0%	0%	0%[a]	39.2% (95% CI: 22.3–55.9%)

[a]One patient in this subgroup (who had 5 cm T2 tumor) developed late local failure at 82 months following chemoradiation.
